# Supplementary material for: Plasma-Induced Surface Modification of Sapphire and Its Influence on Graphene Grown by Plasma-Enhanced Chemical Vapour Deposition
Source: Nanomaterials (Basel). 2023 Jun 27;13(13):1952. doi: 10.3390/nano13131952 (PMC10343755; doi:10.3390/nano13131952)
Supplement: Supplementary file 1 [file nanomaterials-13-01952-s001.zip › nanomaterials-2451307-supplementary.pdf]

Plasma induced surface modification of sapphire and its influence on graphene grown by plasma-enhanced chemical vapour deposition

Authors:

Miguel Sinusia Lozano [1], Ignacio Bernat-Montoya [1], Todora Ivanova Angelova [1], Alberto Boscá Mojena [2], Francisco J. Díaz-Fernández [1], Miroslavna Kovylina [1], Alejandro Martínez[1], Elena Pinilla Cienfuegos [1], Víctor J. Gómez* [1]

1 Nanophotonics Technology Center (NTC), Universitat Politècnica de València, Valencia, Spain

2 Institute of optoelectronic Systems and Microtechnology (ISOM), Universidad Politécnica de Madrid, Madrid, Spain

*E-mail: vjgomher@ntc.upv.es

SUPPLEMENTARY

Study of the synthesis conditions: Plasma power and Temperature combinations

In order to study the influence of the plasma power and temperature during the synthesis of nanographene on sapphire substrates a series of experiments were carried out and the samples were characterized by means of the Raman scattering technique. Before introducing the samples into the synthesis chamber the samples were cleaned using the two-solvent method described in the main document *methods* section.

The synthesis chamber was then heated to the process temperature 800 ºC with a heating rate of 200 ºC/min in a pure Ar atmosphere with a process pressure of 4 mbar and stayed there for 10 minutes. Afterwards, the flow of Ar was halted, and the reactive gas (methane, CH_4) introduced with the following gas ratio (1:5:5, CH_4, N_2 and H_2 respectively). The DC plasma was then turned on with the selected discharge power (Table S 1). The synthesis time was set to 20 min. The system was cooled down at a temperature rate of 15 º/min and samples retrieved at temperatures below 200 ºC.

Table S 1 Synthesis conditions employed for the synthesis of graphene on sapphire substrates.

Temperature (ºC)	Pressure (mbar)	Power (W)	Gases (sccm)	Synthesis time (min)
700	4	50	20 CH_4<br>100 N_2<br>100 H_2	20
800		100		
900		150		

The Raman spectra were fitted using a Lorentzian fitting (Figure S 1) and the position, FWHM, areas and intensities of the fingerprint bands of graphene i.e. D, G, D’, 2D were obtained.

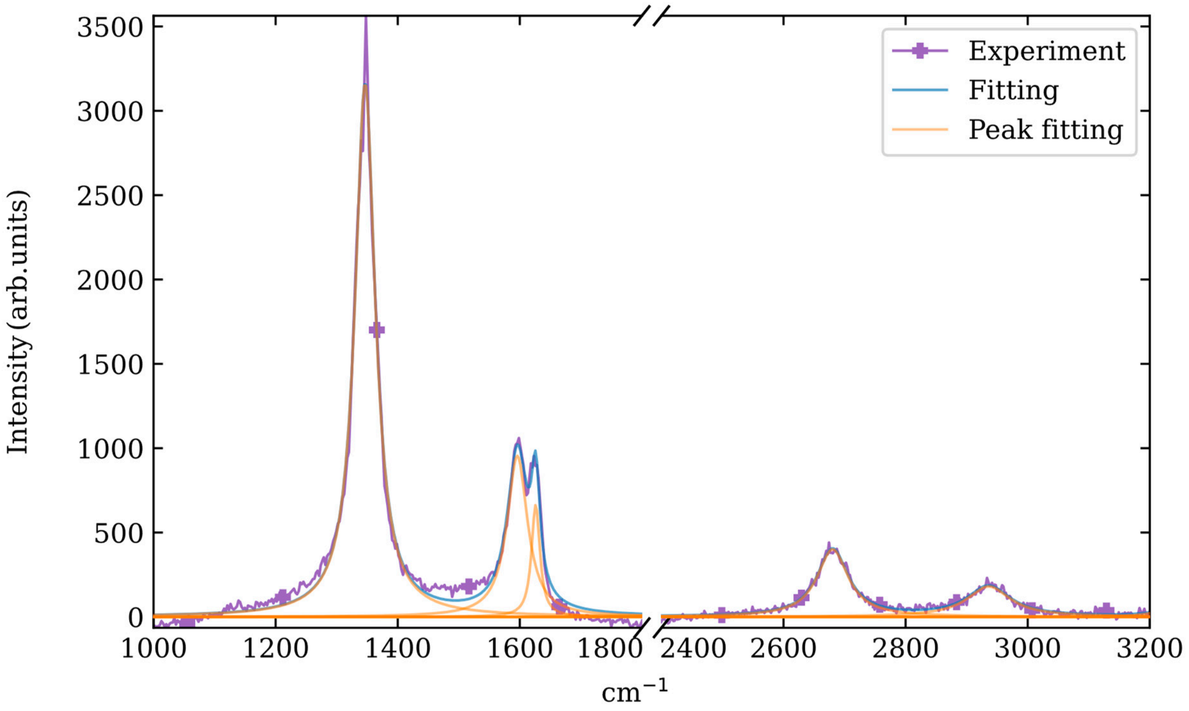

Figure S 1 Lorentzian peak fitting of a Raman spectrum from the synthesized graphene on the reference sample (c-plane sapphire).

The 2D bands are only remarkable in graphene synthesized using 100 W of plasma power (Figure S 2) whereas for other samples the 2D band resembles that of graphite [1].

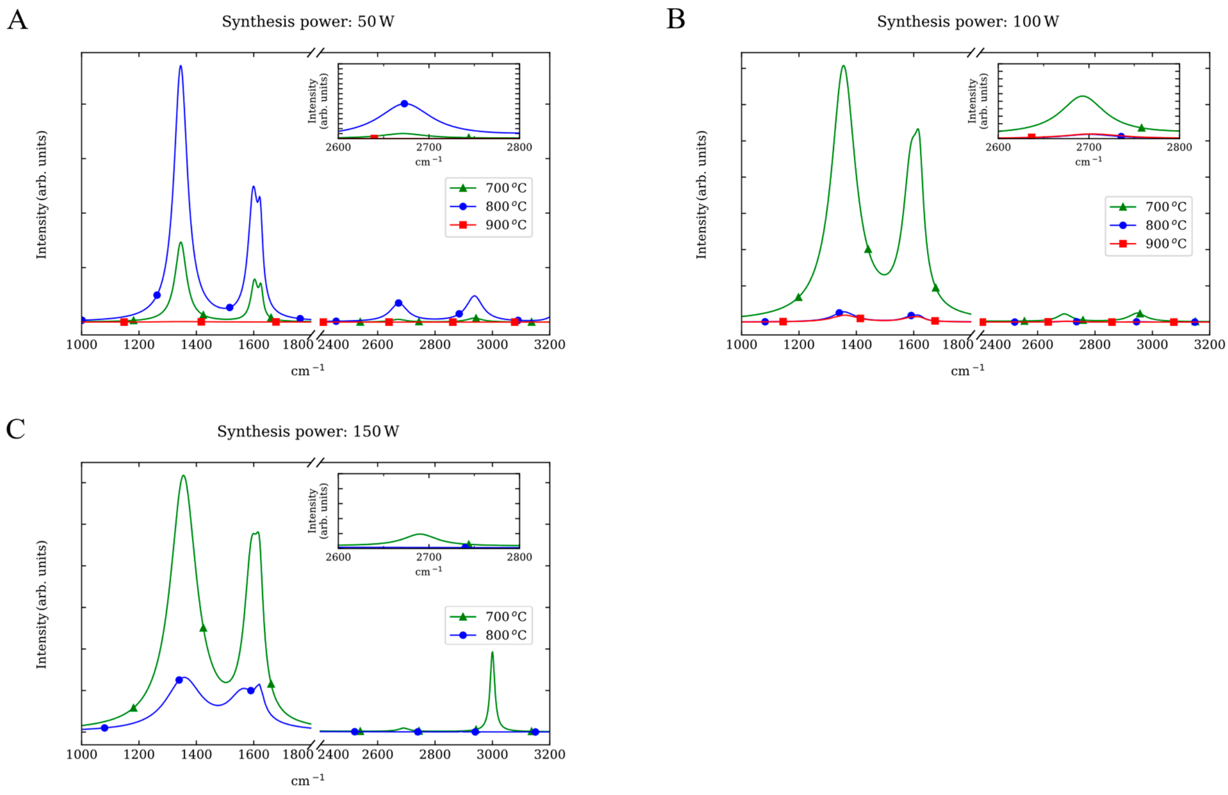

Figure S 2 Raman spectra of graphene synthesized on sapphire with different combinations of plasma power and temperature. A: Synthesis carried out with 50 W B: Synthesis carried out with 100 W C: Synthesis carried out with 150 W.

The intensity ratios provide information about the level of disorder (I_D/I_G) and graphene quality (I_{2D}/I_G) (Figure S 3). The sample grown at 800 ºC and 100 W reports $I_D/I_G = 1.76$ and $I_{2D}/I_G = 0.16$ values together with full width at half maximum values of the G and 2D of 70 cm^{-1} and 69 cm^{-1} respectively. These values, together with the Raman spectrum (Figure S 2B) point to the 800ºC and 100 W combination as the candidate to evaluate the influence of Ar and N_2 etching on the sapphire substrate.

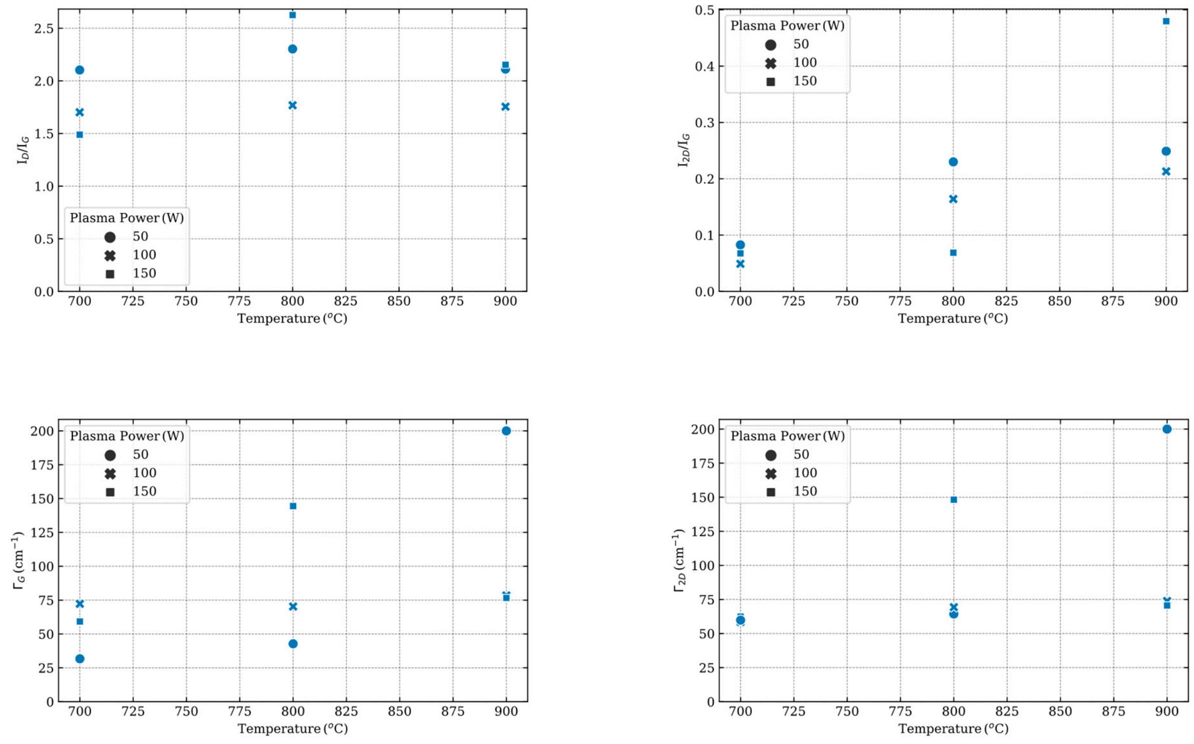

Figure S 3 Influence of the temperature and plasma power employed during the synthesis of graphene on sapphire substrates on the FWHM and on the intensity ratios of the graphene bands in Raman scattering A: I_D/I_G ratio. B: I_{2D}/I_G ratio C: FWHM of G band, D: FWHM of 2D band.

Study of the synthesis conditions: H_2 concentration

In order to study the influence that different H_2 concentrations have on the synthesis of graphene several syntheses were carried (Table S 2). The presence of hydrogen species during the synthesis has been reported to be a major etchant and thus the growing process is a competition between the plasma etching caused by the H species and the growing graphene [2] [3].

Table S 2 Synthesis conditions employed to study the influence of H_2 gas concentration during the synthesis.

Temperature (ºC)	Pressure (mbar)	Power (W)	Gases (sccm)		Synthesis time (min)
				H_2	
800	4	100	20 CH_4 100 N_2	100	20
				180	
				300	

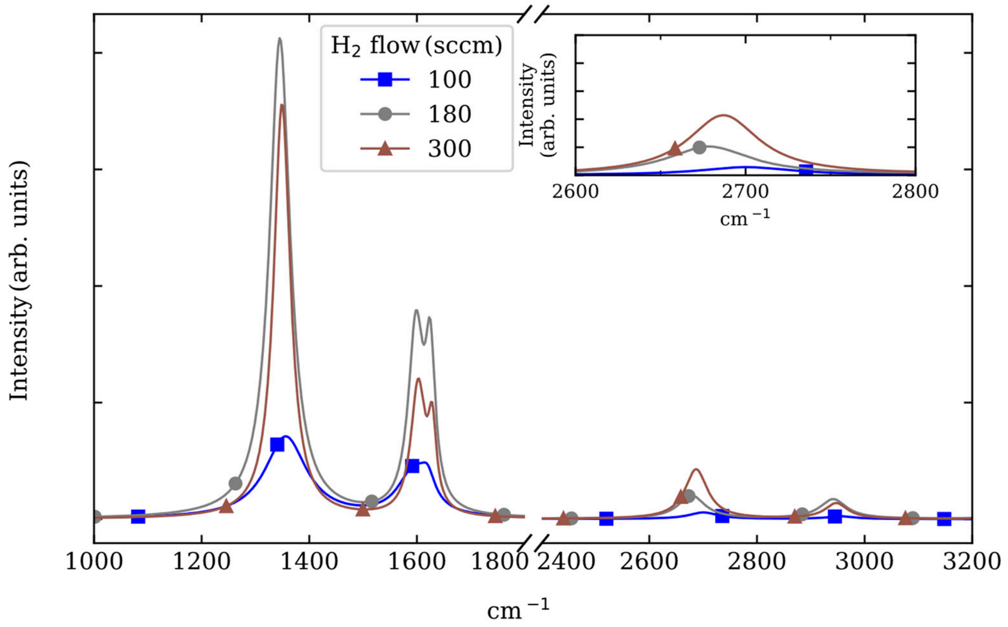

Figure S 4 Raman spectra of graphene samples synthesized on sapphire substrates employing different H_2 flows.

There is a significant increase of the I_{2D}/I_G ratio when the synthesis is carried out using 300 sccm of hydrogen (Figure S 5). However, the I_D/I_G ratio is also increased. On the other hand, the FWHM of the 2D peak is considerably reduced (35 %) indicating that the growth tends to fewer layers of graphene [1]. For that reason, for studying the influence of plasma etching of sapphire substrates in the synthesis of graphene was carried out using the conditions shown in Table S 2 and 300 H_2.

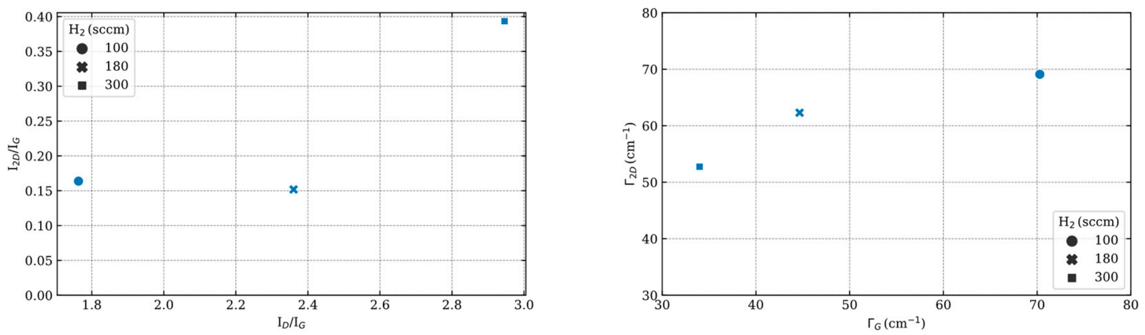

Figure S 5 Raman spectroscopy analysis of graphene synthesized with different H_2 flows A: Ratio of the characteristic Raman bands of graphene: I_{2D}/I_G vs I_D/I_G ratios. B: FWHM values of the 2D and G Raman bands of graphene.

Contact angle measurements

The measurement of the contact angle via the needle-in-drop sessile drop method allows to characterize the wettability properties of a surface. Particularly in c-plane sapphire, the advancing contact angle indicates the surface termination. For a hydrophilic behaviour the surface termination can be ascribed as Al-terminated. On the other hand, the hydrophobic behavior provides larger contact angles, being an indicative of a OH-terminated surface. The drop shapes in the different surfaces (Figure S 6) provide visual evidence of the contact angles that each sapphire and sapphire/graphene surfaces have after the etching and the PECVD synthesis processes.

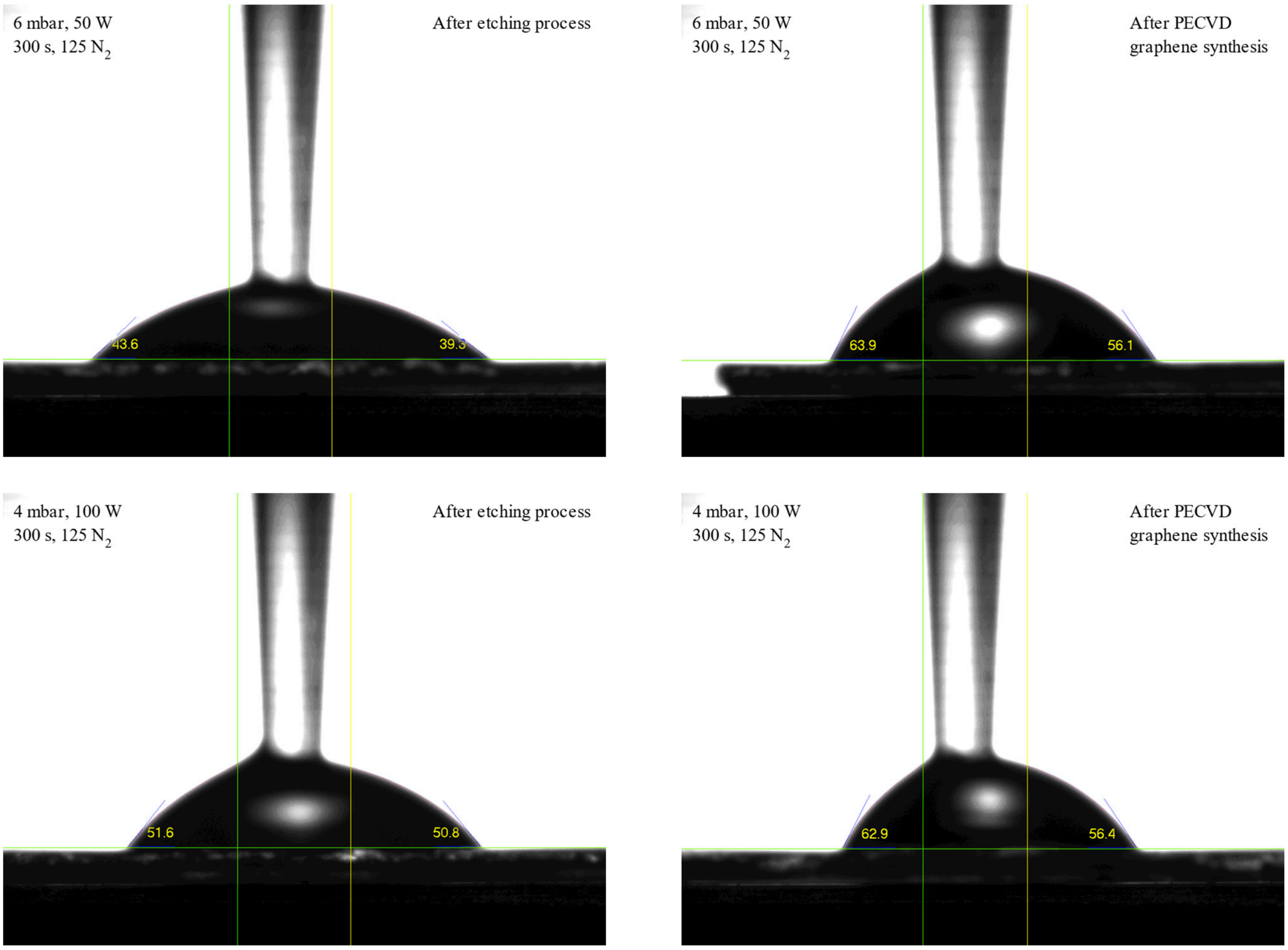

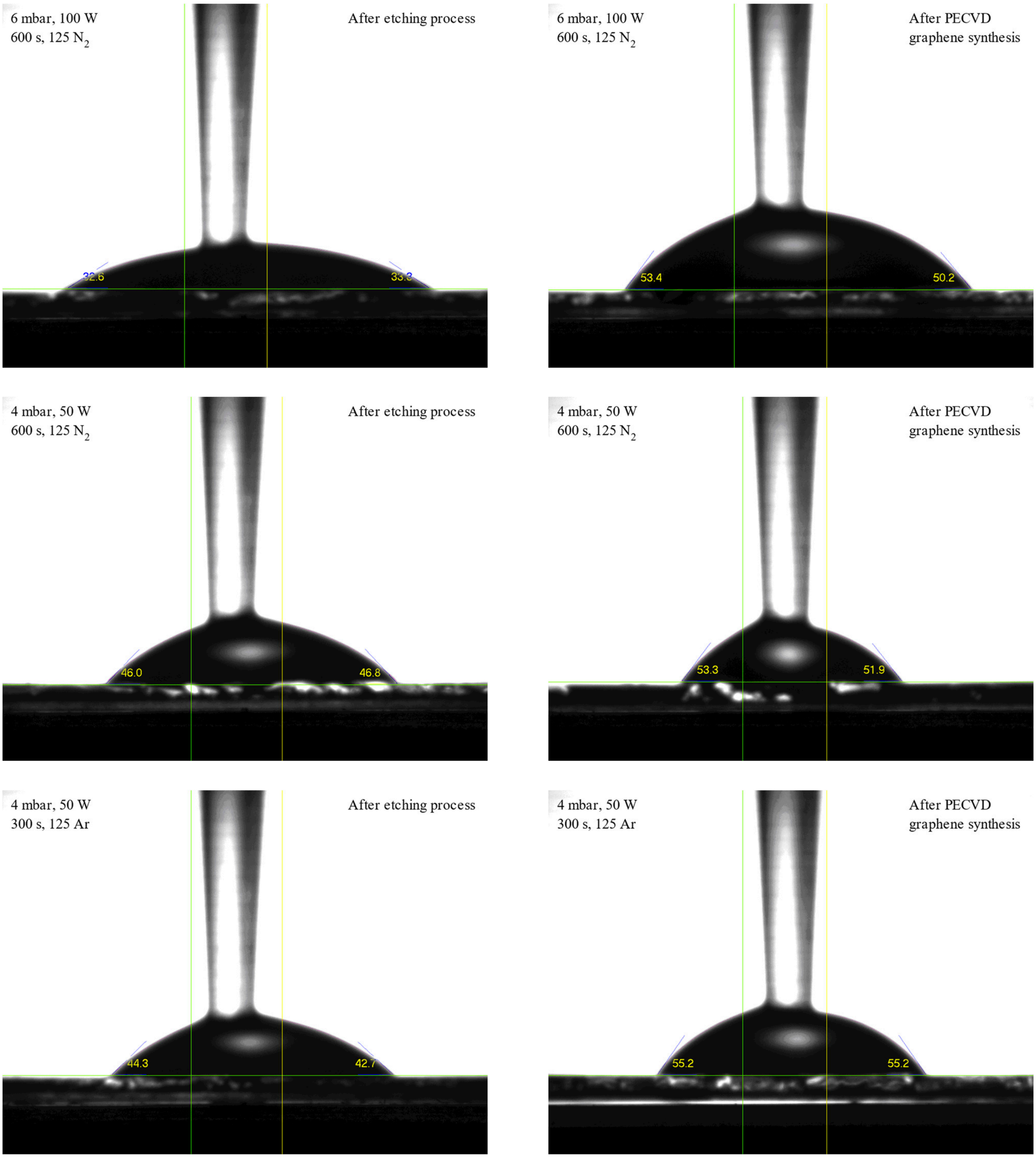
6 mbar, 100 W
600 s, 125 N_2
After etching process
32.6
33.3
6 mbar, 100 W
600 s, 125 N_2
After PECVD
graphene synthesis
53.4
50.2
4 mbar, 50 W
600 s, 125 N_2
After etching process
46.0
46.8
4 mbar, 50 W
600 s, 125 N_2
After PECVD
graphene synthesis
53.3
51.9
4 mbar, 50 W
300 s, 125 Ar
After etching process
44.3
42.7
4 mbar, 50 W
300 s, 125 Ar
After PECVD
graphene synthesis
55.2
55.2

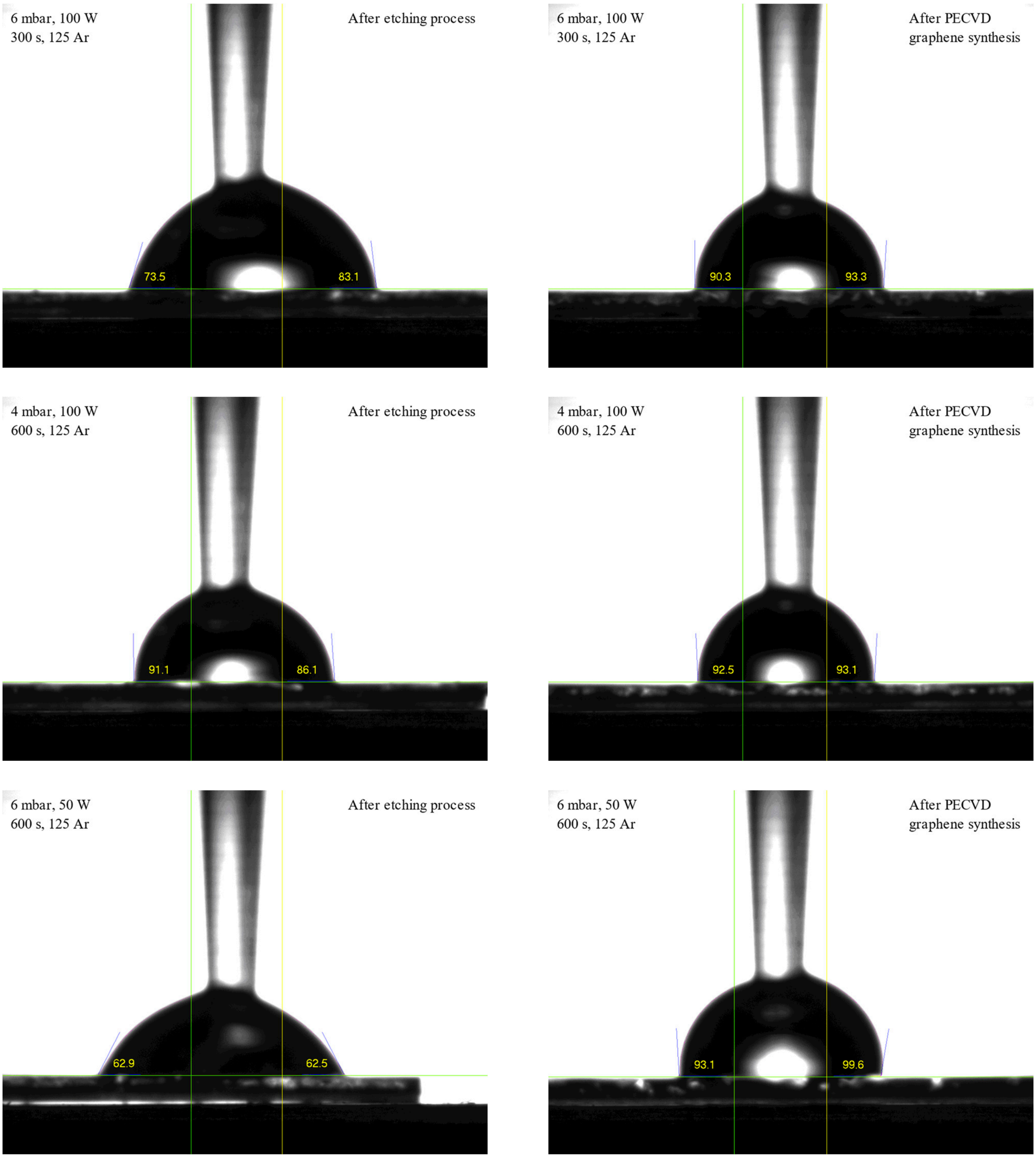
6 mbar, 100 W
300 s, 125 Ar
After etching process
73.5
83.1
6 mbar, 100 W
300 s, 125 Ar
After PECVD
graphene synthesis
90.3
93.3
4 mbar, 100 W
600 s, 125 Ar
After etching process
91.1
86.1
4 mbar, 100 W
600 s, 125 Ar
After PECVD
graphene synthesis
92.5
93.1
6 mbar, 50 W
600 s, 125 Ar
After etching process
62.9
62.5
6 mbar, 50 W
600 s, 125 Ar
After PECVD
graphene synthesis
93.1
99.6

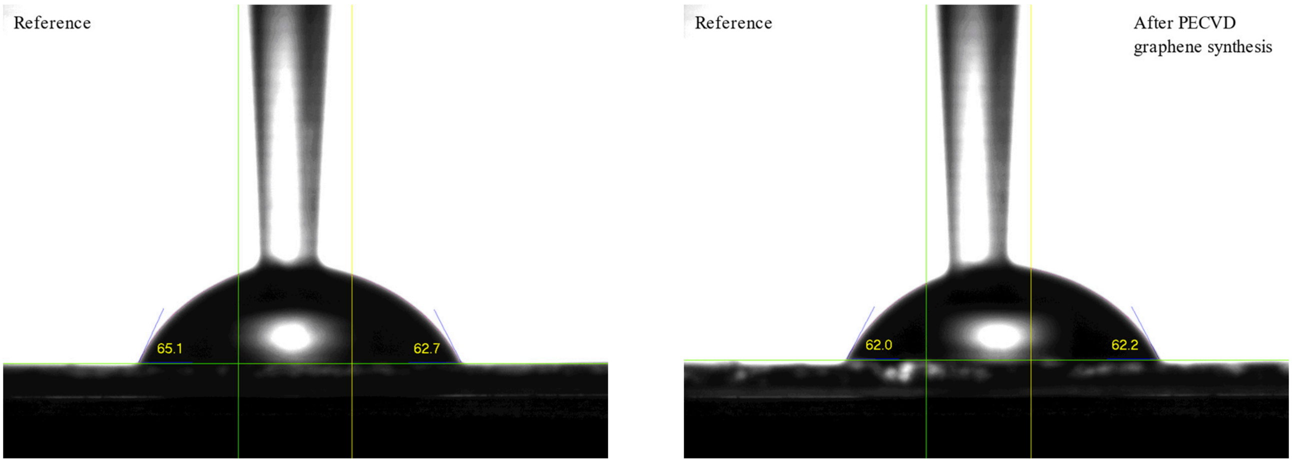

Figure S 6 Pictures of the drop shape during the contact angle measurements. The recipe employed for the etching process is imposed in the upper-left corner of the figures. Left: After carrying out the etching process of sapphire and before the PECVD synthesis of graphene. Right: After the PECVD synthesis of graphene.

Raman spectroscopy: Full coverage analysis

The full coverage of the sapphire substrates is studied via the data dispersion shown by the histograms of the fitted full-width-at-half-maximum (FWHM) values for the graphene D, G and 2D bands (Table S 3). The fitting procedure reports values on the boundaries allowed (i.e. 200 cm-1 for the 2D band FWHM) which allows to discern the presence of the studied band.

Table S 3 Histograms of the FWHM values of the Raman fingerprint bands of Graphene: D, G and 2D bands. Larger values and large scattering of the FWHM are understood as lower coverage or defective graphene.

Sample	Raman band analysis		
	D band	G band	2D band
6 mbar, 100 W, 600 s, N_2	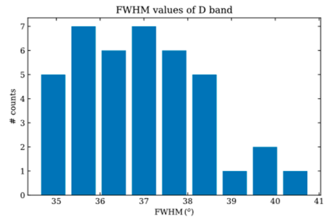	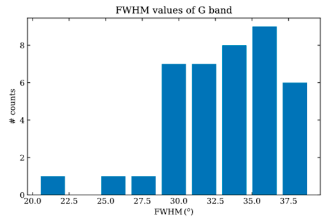	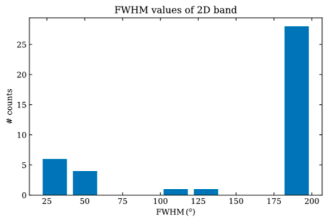
6 mbar, 50 W, 300 s, N_2	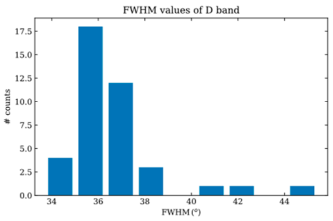	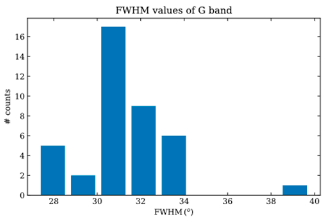	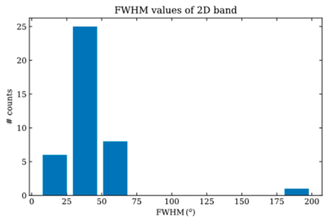
4 mbar, 100 W, 300 s, N_2	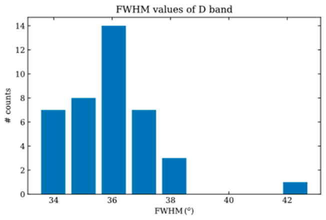	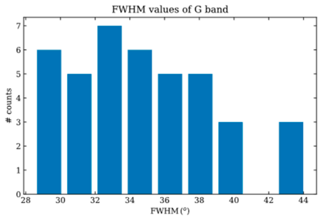	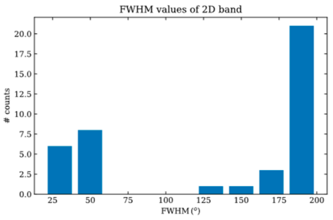
4 mbar, 50 W, 600 s, N_2	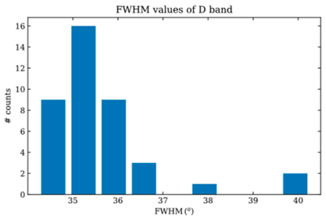	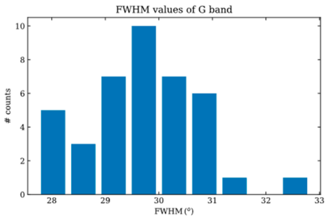	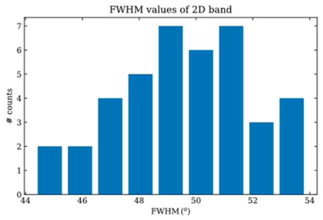

Reference

4 mbar, 50 W,

300 s, Ar

6 mbar, 50 W,

600 s, Ar

6 mbar, 100 W,

300 s, Ar

4 mbar, 100 W,

600 s, Ar

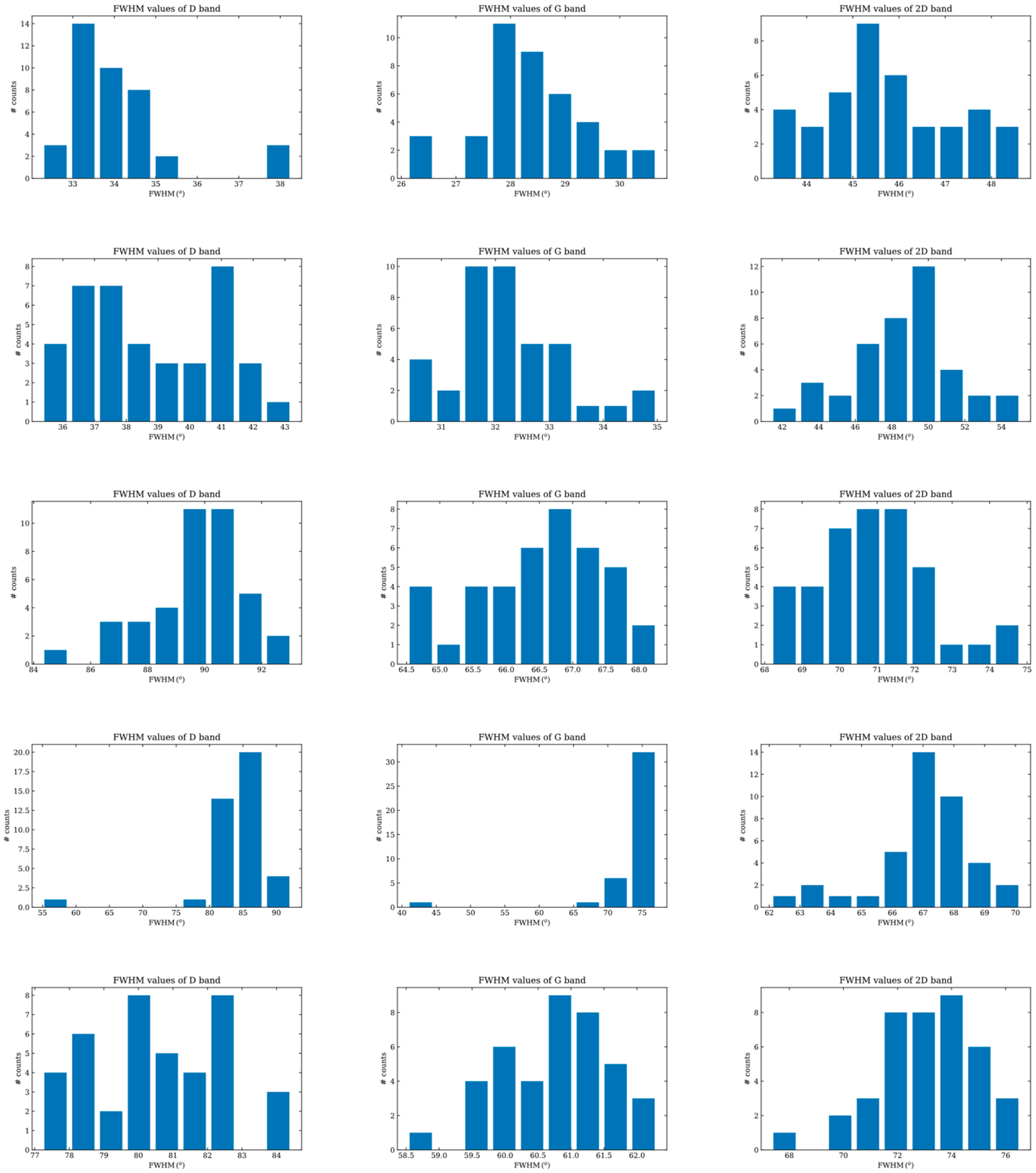

Atomic force microscopy (AFM): Root mean square roughness (R_{RMS})

The etched sapphire substrates were characterised by means of AFM measurements. Their R_{RMS} can be found in Figure S 7.

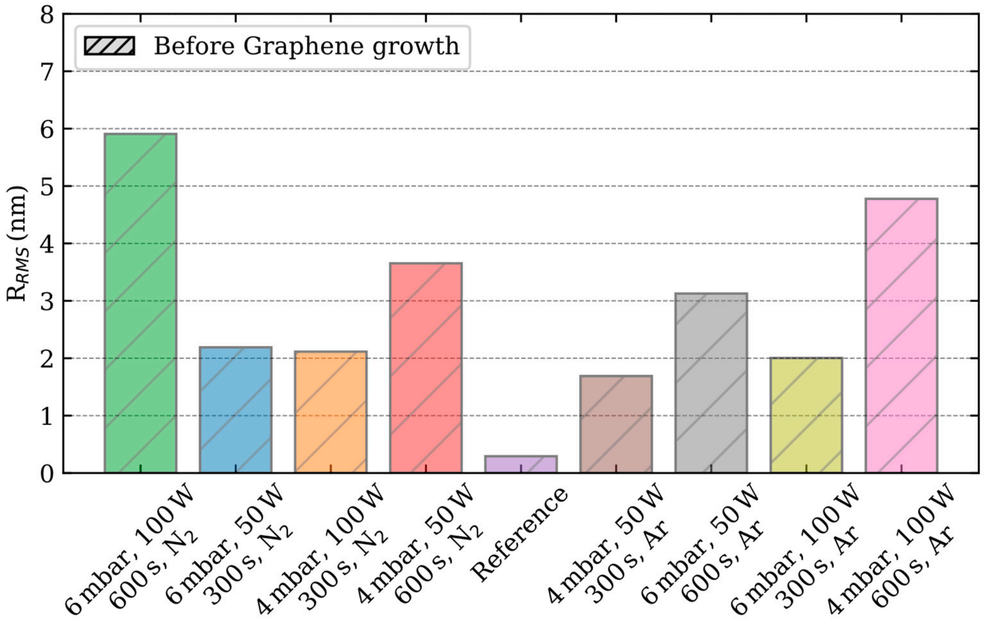

Figure S 7 AFM R_{RMS} values of the sapphire substrates after the plasma processes with different combinations of pressure, discharge power, process time and gas.

Table S 4 Topography, amplitude and phase measurements from the AFM scans of the plasma treatments caried out on the sapphire substrates.

<table>
<tr><th rowspan="2">Sample</th><th colspan="3">AFM scan</th></tr>
<tr><th>Topography</th><th>Amplitude</th><th>Phase</th></tr>
<tr><td>6 mbar, 100 W, 600 s, N_2</td>
<td>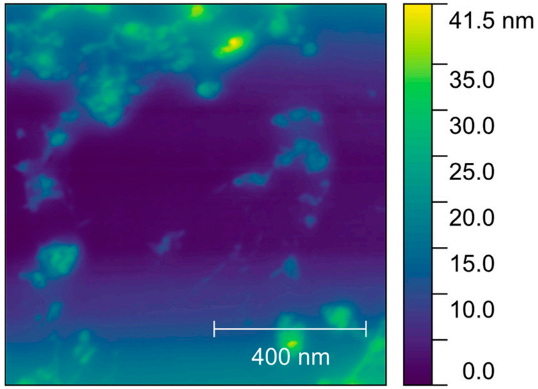
</td>
<td>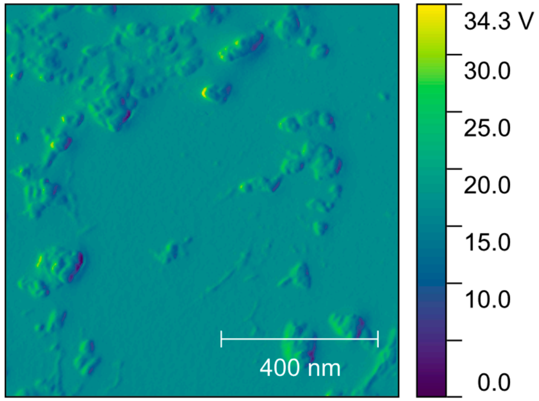
</td>
<td>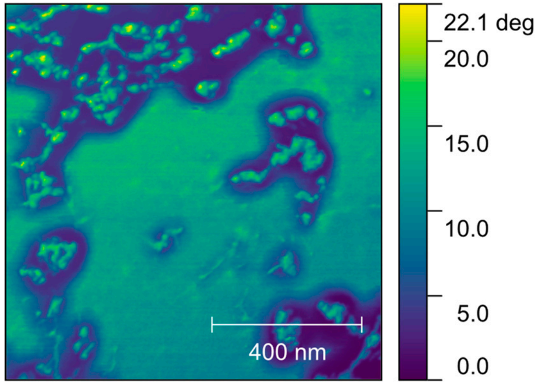
</td></tr>
<tr><td>6 mbar, 50 W, 300 s, N_2</td>
<td>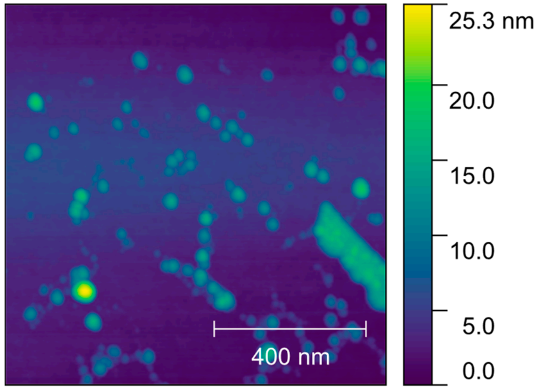
</td>
<td>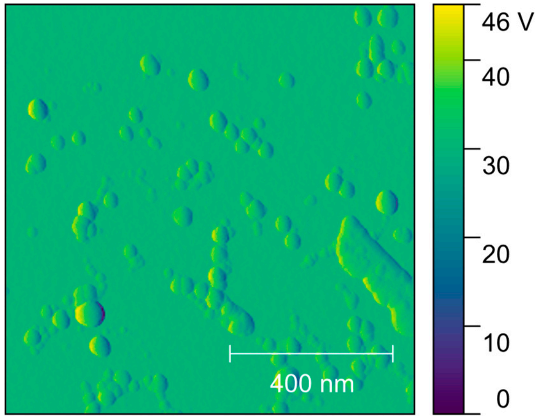
</td>
<td>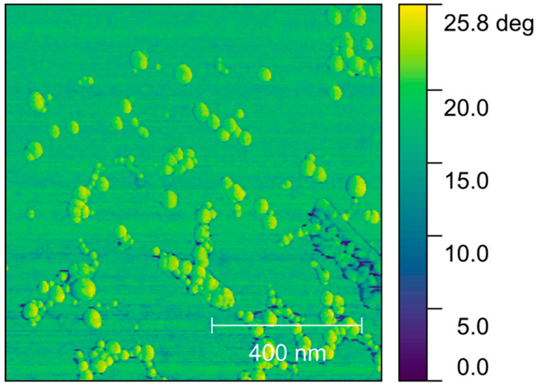
</td></tr>
</table>

4 mbar, 100 W, 300 s, N_2

4 mbar, 50 W, 600 s, N_2

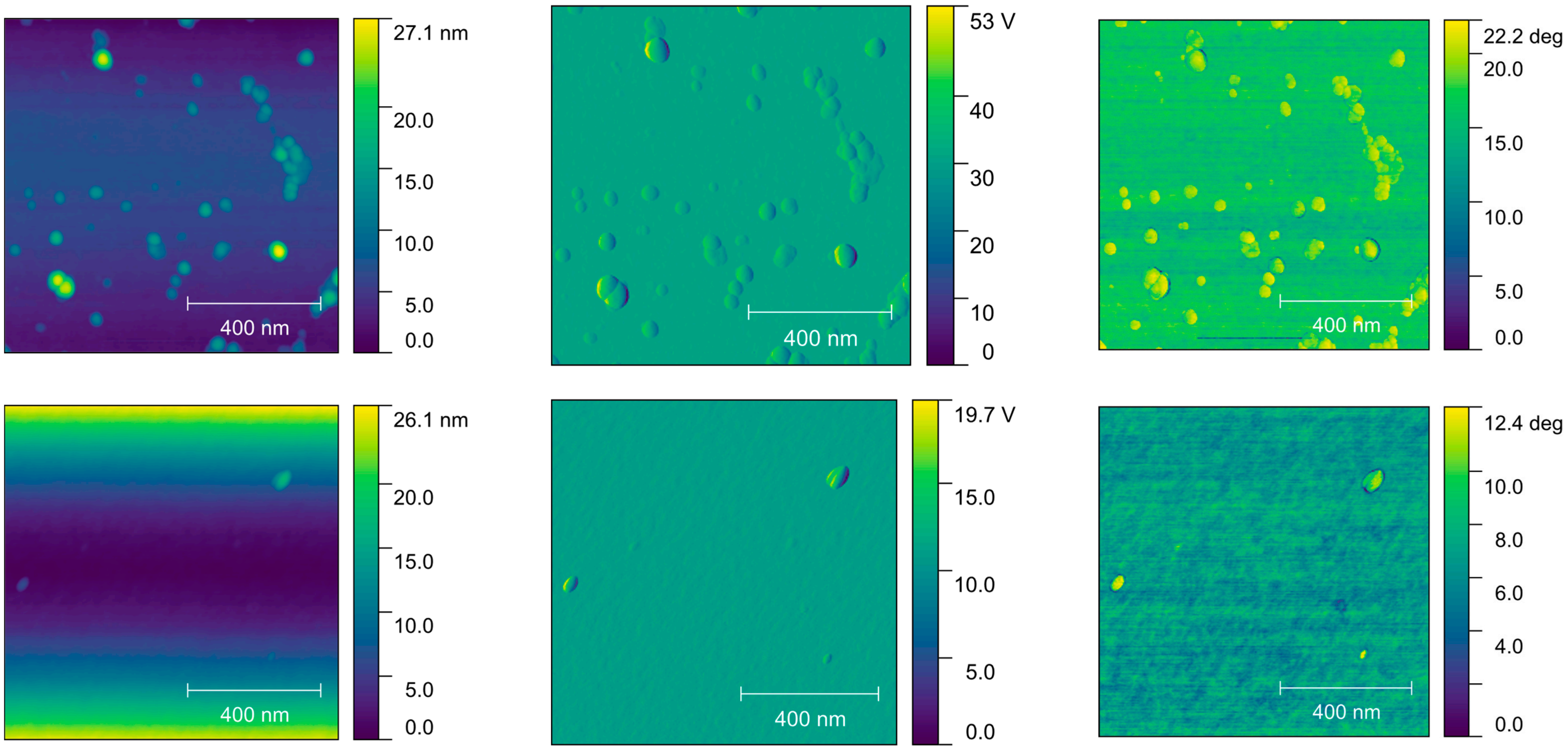

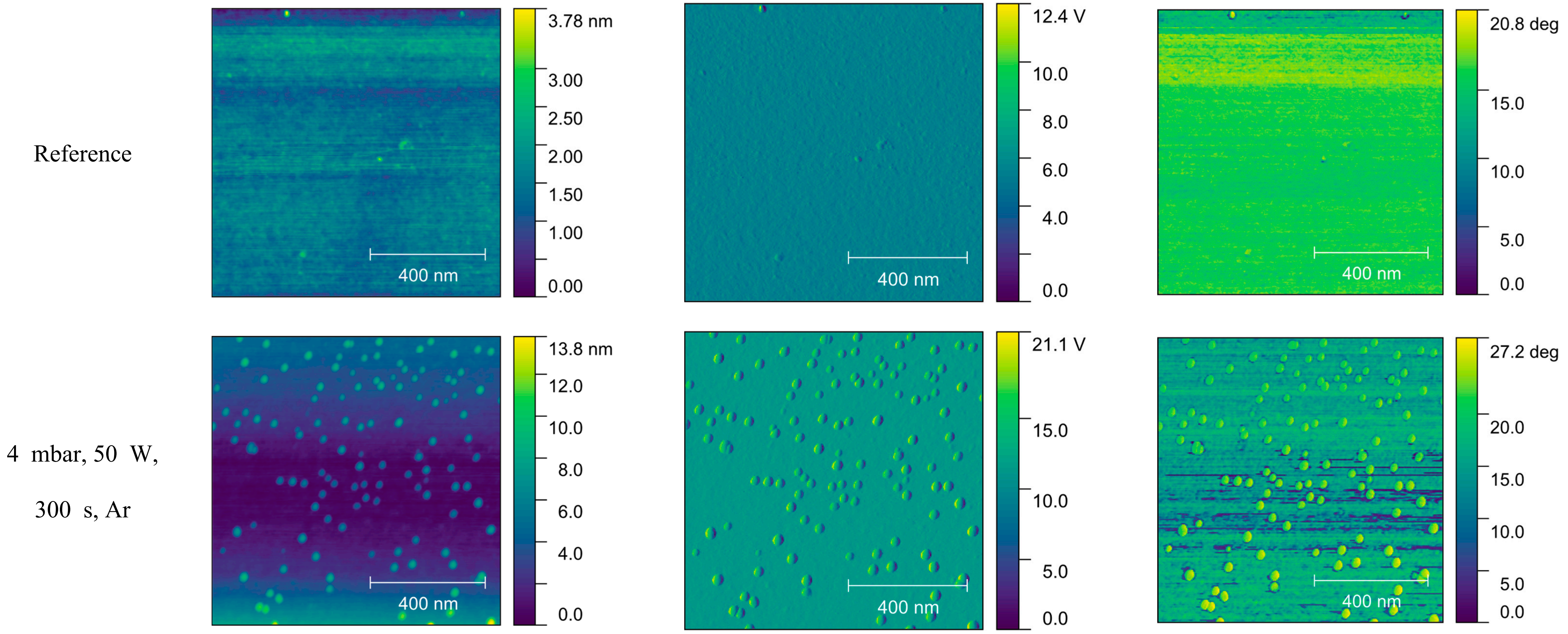

Reference
4 mbar, 50 W,
300 s, Ar
3.78 nm
3.00
2.50
2.00
1.50
1.00
0.00
400 nm
12.4 V
10.0
8.0
6.0
4.0
0.0
400 nm
20.8 deg
15.0
10.0
5.0
0.0
400 nm
13.8 nm
12.0
10.0
8.0
6.0
4.0
0.0
400 nm
21.1 V
15.0
10.0
5.0
0.0
400 nm
27.2 deg
20.0
15.0
10.0
5.0
0.0
400 nm

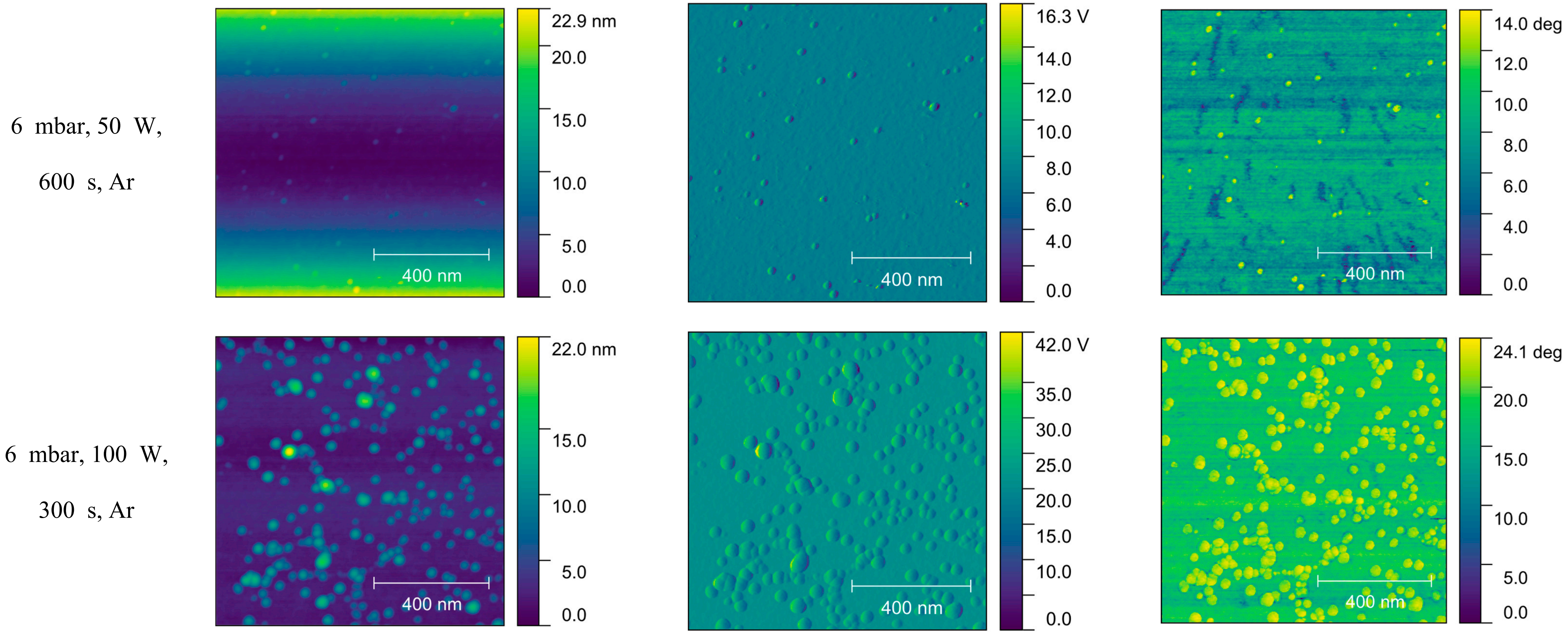
6 mbar, 50 W,
600 s, Ar
6 mbar, 100 W,
300 s, Ar
22.9 nm
20.0
15.0
10.0
5.0
0.0
400 nm
16.3 V
14.0
12.0
10.0
8.0
6.0
4.0
0.0
14.0 deg
12.0
10.0
8.0
6.0
4.0
0.0
22.0 nm
15.0
10.0
5.0
0.0
42.0 V
35.0
30.0
25.0
20.0
15.0
10.0
0.0
24.1 deg
20.0
15.0
10.0
5.0
0.0

4 mbar, 100 W,

600 s, Ar

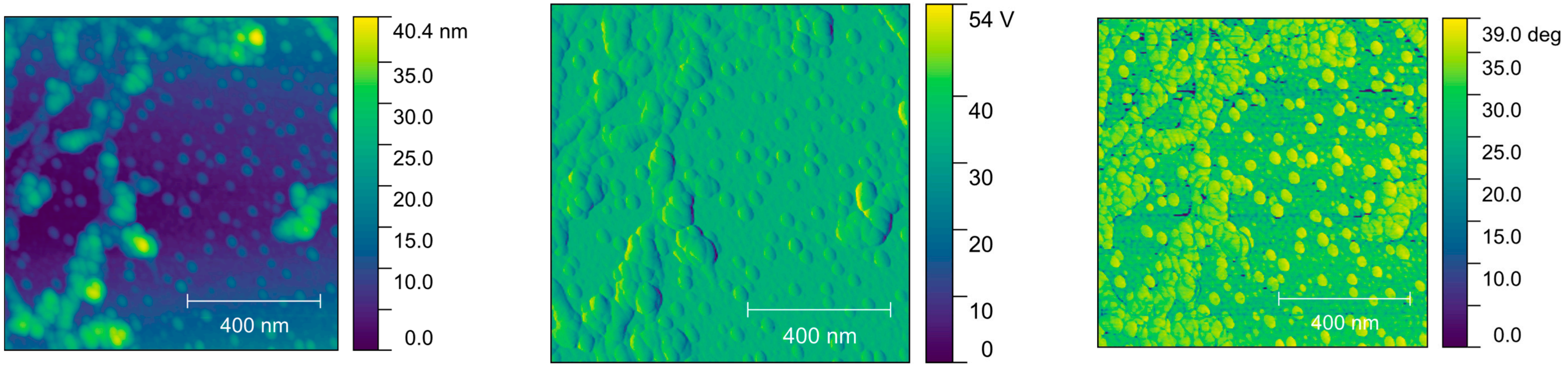

Raman spectroscopy: Laser power influence

The measurement parameters (number of accumulations and acquisition time) were selected in order to increase the counts and the signal-to-noise ratio. For that reason, several measurements at different laser powers for evaluating their influence in the measurements were carried out (Table S 5). Both, the I_D/I_G and I_{2D}/I_G ratios increase when the measurement is carried out at 25 mW as compared to those values obtained when measured at 2 mW. The heating process can be observed due to the shift of the band positions, showing the thermal deformation of the graphene. However, due to the spectral resolution of our system (~3 cm^{-1}) and that the band ratios were not improved, we decided to keep measuring with a laser power of 25 mW (Figure S 8).

Table S 5 Fitting results of the Raman spectroscopy measurements of the synthesized graphene on sapphire measured at two different laser power.

Fitting result	Measurement power: 2 mW	Measurement power: 25 mW
ID/IG	2.04	2.23
I2D/IG	0.32	0.38
D band position	1357 cm^{-1}	1356 cm^{-1}
G band position	1592 cm^{-1}	1588 cm^{-1}
2D band position	2705 cm^{-1}	2700 cm^{-1}

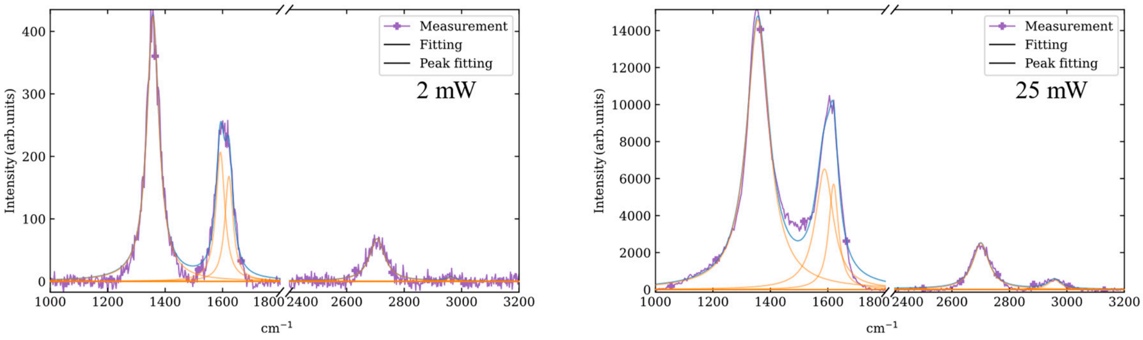

Figure S 8 Raman measurement of graphene: Left: 2 mW. Right: 25 mW.

Another process we were concerned was the oxidation process that could be promoted because of the large laser power employed. However, we did not observe any oxidation process, carrying out 100 measurements in the same spot, without deflecting the laser (meaning constant irradiance) every 2 seconds (2 s of integration time) (Figure S 9).

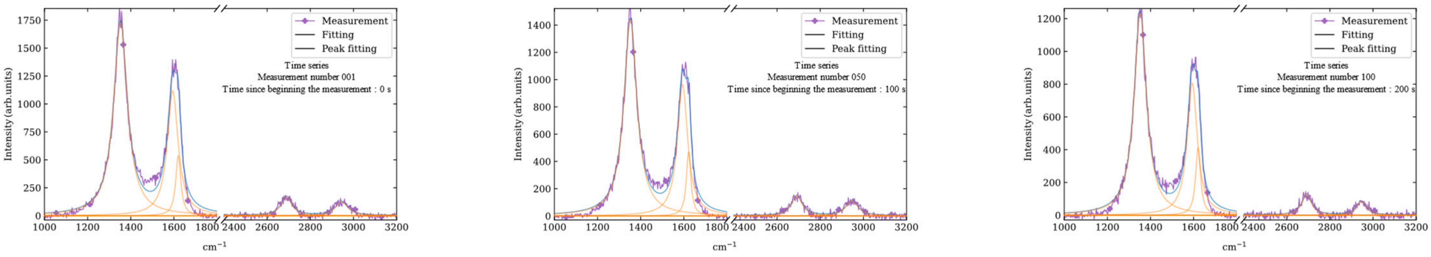

Figure S 9 Raman spectra at different times of laser irradiance. Left: Measurement time: 0s. Middle: Measurement time: 100 s. Right: Measurement time 200 s.

REFERENCES

[1] L. M. Malard, M. A. Pimenta, G. Dresselhaus, and M. S. Dresselhaus, 'Raman spectroscopy in graphene', *Phys. Rep.*, vol. 473, no. 5, pp. 51–87, Apr. 2009, doi: 10.1016/j.physrep.2009.02.003.

[2] R. Gudaitis, A. Lazauskas, Š. Jankauskas, and Š. Meškinis, 'Catalyst-Less and Transfer-Less Synthesis of Graphene on Si(100) Using Direct Microwave Plasma Enhanced Chemical Vapor Deposition and Protective Enclosures', *Materials*, vol. 13, no. 24, p. 5630, Dec. 2020, doi: 10.3390/ma13245630.

[3] I. Vlassiouk *et al.*, 'Role of Hydrogen in Chemical Vapor Deposition Growth of Large Single-Crystal Graphene', *ACS Nano*, vol. 5, no. 7, pp. 6069–6076, Jul. 2011, doi: 10.1021/nn201978y.
